# Negative affectivity moderates the association between job demands and hair cortisol in healthcare workers: a cross-sectional multi-method study

**DOI:** 10.3389/fpsyg.2026.1740056

**Published:** 2026-10-30

**Authors:** Elvira Arcucci, Alessandra Falco, Sebastiano Rapisarda, Laura Dal Corso, Isabella Pividori, Damiano Girardi

**Affiliations:** 1 FISPPA Section of Applied Psychology, University of Padova, Padova, Italy; 2 Department of Agricultural, Environmental and Animal Sciences, University of Udine, Udine, Italy

**Keywords:** hair cortisol, healthcare, job demands, negative affectivity, work-related stress

## Abstract

**Aims:**

Healthcare workers (HCWs) are frequently exposed to psychosocial job demands (JDs) that may lead to work-related stress. This study investigates the association between JDs—specifically time pressure, problem solving, overload, and emotional demands—and hair cortisol concentration (HCC), as biomarker of long-term hypothalamic–pituitary–adrenal axis activation, in a sample of HCWs. It further examines the moderating role of negative affectivity (NA), an individual characteristic linked to heightened stress reactivity. Drawing on the Job Demands–Resources theory and the allostatic load model, we hypothesized that (1) JDs would be positively associated with HCC and (2) this relationship would be stronger for individuals high in NA.

**Design:**

Participants were 67 healthcare professionals working in a healthcare facility located in Northern Italy. Participants completed a self-report questionnaire designed to assess JDs and NA. Next, participants provided a hair sample for the assessment of HCC.

**Results:**

Although data from a sample of HCWs revealed some positive but non-significant trends for problem solving and overload, generally JDs were not associated with HCC at average levels of NA. Interestingly, NA moderated the relationship between problem solving/time pressure and HCC, with stronger positive associations among individuals with high NA.

**Conclusion:**

Findings from this exploratory cross-sectional study suggest that NA may act as a vulnerability factor, implying that HCWs high in NA may have a heightened strain response to certain JDs. By integrating psychological and biological measures, this study offers novel insights into the interplay between workplace stressors, personality traits, and physiological responses to stress. These findings highlight the potential relevance of individual differences in occupational stress research, and point to HCC as a promising indicator of chronic stress among HCWs.

**Implications for the profession:**

The findings suggest the need for occupational health interventions tailored to individual traits, such as NA, to reduce stress reactivity, foster well-being, and enhance the quality of care provided.

## Introduction

Healthcare is widely recognized as a high-risk sector for work-related stress (W-RS) and psychophysical strain, with employees exposed to numerous threats to their health and well-being (Lin et al., 2025; Marzocchi et al., 2024). Healthcare workers (HCWs) face different psychosocial risks, including excessive workloads, high emotional and cognitive demands, time pressure, irregular working hours, hostile social interactions (e.g., violence, harassment, and bullying), and insufficient organizational support (Adriaenssens et al., 2015; Broughton et al., 2025). These conditions not only endanger the health of HCWs but also compromise the quality of care provided (Broughton et al., 2025).

Although W-RS has been extensively studied among HCWs, previous research has predominantly relied on self-report measures to examine the association between job demands (JDs) and strain responses. Accordingly, there is a growing need to incorporate objective indicators that can complement and validate subjective assessments (Bakker and Demerouti, 2017). In this context, stress biomarkers offer a promising avenue for assessing the physiological impact of chronic stress. Among these, hair cortisol concentration (HCC) has received increasing attention as a reliable and non-invasive indicator of long-term cortisol accumulation, reflecting sustained exposure to stress—even in occupational healthcare settings (Bakker and Demerouti, 2017; Schaafsma et al., 2021).

However, past research on W-RS and HCC presents mixed findings (Rohleder, 2018; Schaafsma et al., 2021). These inconsistencies may be partly attributable to the fact that individual characteristics influencing the strain response have not always been sufficiently considered in the literature. Specifically, drawing on transactional models of stress, individual differences appear to play a crucial role in shaping how stressors are appraised and managed (e.g., Bongelli et al., 2021; Martos Martínez et al., 2021).

Against this background, this study examined whether the association between JDs commonly experienced in healthcare and HCC differed across levels of NA, offering new insights into the role of individual differences in the link between psychological and physiological stress responses.

## Theoretical background

In the context of the Job Demands–Resources (JD-R) theory proposed by Demerouti et al. (2001), JD refer to the physical and/or psychological aspects of a job that require effort and are linked to certain physiological or psychological costs. Prolonged or chronic exposure to high levels of JDs can result in exhaustion and various health complaints over time (Nixon et al., 2011; Schaufeli and Taris, 2014). Notably, JDs may reflect the physical, psychological, social, or organizational nature of work, and in the healthcare sector include aspects such as workload, overload, and emotional demands (Broughton et al., 2025; Falco et al., 2018; Peter et al., 2024; Karasek et al., 1998).

In the literature on W-RS, workload is usually considered as a psychological JD (Karasek et al., 1998) that is typically described along two related dimensions: quantitative workload, referring to the amount of work expected within a given timeframe, and qualitative workload, referring to the complexity or difficulty of tasks that may exceed employees’ skills or available resources (Bowling and Kirkendall, 2012). In the present study, these dimensions are operationalized as time pressure and problem solving, respectively (De Carlo et al., 2019). Both dimensions of workload were associated in the literature with psychophysical strain (Demerouti et al., 2001; Kubicek et al., 2023).

Moreover, work overload is a specific manifestation of a quantitative workload that is typically defined as “the extent to which job performance requirements are excessive or overwhelming” (Iverson and Maguire, 2000). This definition highlights a volume of tasks that exceeds employees’ capacity to perform them within the available time and conditions. Notably, work overload is one of the most commonly measured occupational stressors, and it has been shown to be associated with elevated self-reported stress and physiological strain responses in occupational settings (Mander and Antoni, 2022).

Finally, work in health and social care is generally characterized as being emotionally demanding (Bakker and Demerouti, 2017). Emotional demands refer to emotionally charged interactions at work (e.g., with patients and/or families; Xanthopoulou et al., 2013). Accordingly, emotionally demanding working conditions require an investment of energy that may deplete employees’ resources and are considered an important source of job strain (Marzocchi et al., 2024).

The first aim of this study is to investigate the association between perceived JDs (i.e., psychological data) with biological data in terms of HCC as a possible biomarker of W-RS (Schaafsma et al., 2021). The use of HCC is particularly relevant, as it reflects the cumulative activation of the HPA axis, which is considered one of the most important biological systems involved in the strain response (Rohleder, 2018; Schaafsma et al., 2021).

### Hair cortisol as a biomarker of stress

The allostatic load (AL) model (McEwen, 1998) offers an integrative theoretical framework to explain the association between perceived JDs and long-term effects of W-RS on physical and mental health via cumulative dysregulation in the HPA axis, as reflected by HCC (Juster et al., 2010). According to this model, repeated or prolonged exposure to JDs can result in sustained activation of the body’s strain response systems, including the secretion of stress hormones such as cortisol (van der Meij et al., 2018; Girardi et al., 2025). Over time, especially in the absence of adequate recovery (Geurts and Sonnentag, 2006), this sustained activation may result in dysregulation of biological systems—including the HPA axis—and contribute to negative health outcomes (Bellingrath et al., 2008; Juster et al., 2010; Zänkert et al., 2019). In summary, by reflecting the cumulative concentrations of cortisol over extended periods, HCC provides a reliable marker of prolonged HPA axis activation and can be considered a biomarker of W-RS in the AL framework (Schaafsma et al., 2021).

In line with this reasoning, we hypothesize that the exposure to JDs—including workload, overload, and emotional demands—will be associated with higher HCC, indicating dysregulation of the HPA axis in the context of W-RS. This is consistent with previous research showing a positive association between JDs and HCC (for a review see Schaafsma et al., 2021).

*Hypothesis 1 (H1)*. Job demands (in terms of time pressure, problem solving, overload, and emotional demands) will be positively associated with hair cortisol concentration.

### The role of negative affectivity

Transactional models of stress (Lazarus and Folkman, 1984) conceptualize W-RS as a process in which perceived characteristics of the work environment deplete individual resources and elicit coping responses, ultimately leading to psychological, physiological, and behavioural outcomes that are associated with both short- and long-term changes in health (Hart and Cooper, 2001; Ganster and Rosen, 2013; Nixon et al., 2011). Individual characteristics such as negative affectivity (NA) play a key role in this process, since they may affect how JDs are appraised and managed, as well as their impact on the strain response (Bakker and Demerouti, 2017).

As posited by Watson and Clark (1984), NA is a dispositional dimension that reflects pervasive individual differences in the conception of the self and the tendency to experience aversive emotional states. Individuals with high levels of NA also tend to hold a negative view of themselves, their environment, and the world in general, perceiving it as hostile and threatening. They have also been observed to report elevated levels of psychological distress, reduced self-satisfaction, and diminished task performance and organizational citizenship behaviors (Kaplan et al., 2009; Thoresen et al., 2003; Watson et al., 1999).

Although there is extensive debate in the literature on the role of NA in the stress process (Brief et al., 1988; Falco et al., 2013; Lazuras et al., 2009; Oliver et al., 2010; Spector et al., 2000), past research suggests that it can affect the reactivity to stressful events, or “the extent to which an individual is likely to show emotional or physical reactions to a stressful event” Bolger and Zuckerman (1995, p. 890). Spector et al. (2000) referred to this mechanism as the “hyper-responsivity mechanism.” In this perspective, high NA individuals may react more negatively to JDs, with more negative strain response compared to those with low NA (Spector et al., 2000). Stated differently, NA may affect outcomes of WR-S by enhancing (i.e., moderating) the effects of JDs on these outcomes (Bolger and Zuckerman, 1995).

Building on the conceptualization of NA as an individual characteristic that influences stress reactivity, we hypothesize that NA may strengthen the positive association between JDs—in terms of time pressure, problem solving, overload, and emotional demands—and HCC.

*Hypothesis 2 (H2)*: Negative affectivity will moderate the association between job demands (in terms of time pressure, problem solving, overload, and emotional demands) and HCC, which is expected to be stronger for individuals with a high level of NA.

## Methods

### Procedure and participants

Participants were healthcare professionals working in a healthcare facility located in Northern Italy, who were free and autonomous in deciding whether or not to take part in the study. The research was structured in two phases, aimed, respectively, at collecting psychological and biological data. In the first phase, participants completed a self-report questionnaire designed to assess psychological data, in terms of JDs and NA. Each participant entered a unique alphanumeric identifier at the beginning of the questionnaire, which enabled matching the psychological data with the biological data collected in the second phase. In this second phase, participants provided a hair sample for the assessment of HCC, serving as the biological indicator.

Prior to participating in the study, each participant was informed about the research objectives and assured that participation was voluntary and confidential. They were made aware of their right to withdraw from the study at any time, without providing an explanation, without penalty, and with the guarantee that their data would not be used. Written informed consent was obtained from all participants before data collection. The study was performed in line with the principles of the Declaration of Helsinki. The project was approved by the Psychological Research Ethics Committee (Area 17), Department/Section of Psychology - University of Padua, Italy (protocol n. 4,072).

A total of 96 workers completed the psychological questionnaire, whereas 67 participants completed both psychological and biological data collection and were thus considered the final sample for the study. Workers with adrenocortical dysfunction or using glucocorticoid drugs were excluded from the study. Data on the use of hair cosmetic treatments (e.g., hair dyeing, bleaching, or frequent chemical treatments) were not collected in the present study.

No significant differences were found in sociodemographic or focal study variables between those who fully participated and those who did not (i.e., those who completed only the self-report questionnaire). The final sample consisted of 67 participants, including 55 females (82.1%) and 12 males (17.9%). Regarding occupational roles, 56 participants were nurses (83.6%) and 11 physicians (16.4%). Most participants held a permanent employment contract (95.5%) and reported having parental responsibilities (56.7%).

### Psychological measures

To assess psychological variables, participants completed a self-report questionnaire comprising the following standardized measures:

Job demands were measured using a scale taken from the Q_u_-Bo Test, an instrument standardized for the Italian context (De Carlo et al., 2008). The scale included 12 items designed to assess problem solving (three items, “constantly solve new problems”) and time pressure (four items, “work very fast”)—reflecting qualitative and quantitative workload, respectively (De Carlo et al., 2019)—as well as overload (two items, “excessive amount of overtime work”) and emotional demands (three items, “emotionally demanding work situations”). In this study, Cronbach’s alpha was 0.84 for problem solving, 0.72 for time pressure, 0.78 for overload and 0.70 for emotional demands.

Negative affectivity was measured using a short form of the Strain-Free Negative Affectivity Scale, adapted for the Italian context (Girardi et al., 2011). A sample item is “When I am assigned a difficult project, I am very worried”. In this study, Cronbach’s alpha was 0.64, a value considered within the lower limit of acceptability (Hair et al., 2019). A robustness check using a three-item version of the scale is reported in the Model Diagnostics and Sensitivity Analyses section.

The 6-point response scale ranged from 1 (*strongly disagree*) to 6 (*strongly agree*), with higher scores indicating high levels of JDs and NA.

### Biological measures

Hair samples were collected in a painless and non-invasive manner from the vertex posterior region of the scalp. They were stored at room temperature inside paper envelopes and shielded from UV exposure until further processing. The full procedure is detailed in Falco et al. (2023). In summary, twenty-five milligrams of hair were weighed, and each strand was washed twice using H2O (3 min each) and twice with isopropanol (3 min each), following the protocol by Davenport et al. (2006). Cortisol extraction involved incubating the samples in methanol at 37 °C for 16 h. The resulting solution was then evaporated to dryness at 37 °C using an airstream suction hood. The dry residue was reconstituted in 1.2 mL of ELISA buffer (50 mM phosphate buffer, pH 7.4, 0.4% BSA, 0.5 M NaCl). Cortisol concentrations were subsequently quantified using an in-house Enzyme-linked Immunosorbent Assay (ELISA) method (Falco et al., 2023).

### Data analysis

The proposed relationships were examined through moderated multiple regression analyses. Four distinct models were estimated. In Model 1 (M1), HCC were regressed on problem-solving, negative affectivity, and their interaction. The structure of the remaining models was identical, except that Model 2 (M2) included time pressure as the job demand, while Model 3 (M3) included overload, and Model 4 (M4) included emotional demands. All predictor variables in Models 1 through 4 were mean-centered to aid in the interpretation of the coefficients. When significant interaction effects were detected, simple slope analyses were conducted to evaluate the relationship between the specific job demand and HCC at high (+1 *SD*) and low (−1 *SD*) levels of NA. Graphical representations of significant interactions were also provided (Cohen et al., 2003). Each model controlled for sex (Dettenborn et al., 2012), in light of documented sex differences in HCC reported in the literature (Binz et al., 2018; Stalder et al., 2017). Similarly, previous studies have identified variations in health-related stress outcomes—such as job burnout and sleep quality—across different occupational roles in the healthcare sector (Carmassi et al., 2022; Peter et al., 2020). Therefore, occupational role was also included as a control variable. Lastly, a natural logarithmic transformation was applied to HCC to better approximate a normal distribution (Becker et al., 2019).

## Results

### Descriptive statistics

The descriptive statistics and correlations are presented in Table 1. All psychological variables and log HCC had univariate skewness and kurtosis that fell within the acceptable range of ±2.0 and ±7.0, respectively. There was a positive, small- to medium-sized correlation (Cohen et al., 2003) between overload and HCC, *r*_65_ = 0.26, *p* = 0.03, while the association between problem solving and HCC was positive but marginally significant, *r*_65_ = 0.22, *p* = 0.07. Negative affectivity was positively associated with time pressure, *r*_65_ = 0.35, *p* < 0.01, and emotional demands, *r*_65_ = 0.26, *p* = 0.04.

**Table 1:** Means, standard deviations, and correlations (*N* = 67).

Variable	*M*	*SD*	1	2	3	4	5	6
1. Hair cortisol (pg/mg)	12.82	14.24	−					
2. Problem solving	4.00	1.19	0.22†	(0.84)				
3. Time pressure	4.24	1.00	0.07	0.57**	(0.72)			
4. Overload	3.45	1.60	0.26*	0.57**	0.53**	(0.78)		
5. Emotional demands	3.60	1.41	0.10	0.46**	0.39**	0.54**	(0.70)	
6. Negative affectivity	3.60	0.90	−0.02	0.10	0.35**	0.14	0.26*	(0.64)

M and SD are used to represent mean and standard deviation, respectively. Cortisol values were log-transformed prior to data analyses, including correlations shown above. Internal consistency of the scales (Cronbach’s alpha) is displayed in the diagonal of the correlation matrix within parentheses.

^†^*p* < 0.10. *p < 0.05. **p < 0.01.

With respect to control variables, a sex difference emerged in overload, *t*(16) = −2.16, *p* = 0.046, Cohen’s *d* = −0.69, with male participants reporting higher scores. Similarly, significant differences by occupational role emerged for problem solving, *t*(17.7) = −3.53, *p* < 0.01, Cohen’s *d* = −1.05, and overload, *t*(18) = −3.93, *p* < 0.01, Cohen’s *d* = −1.16, while marginally significant differences were observed for log HCC, *t*(11.1) = −1.81, *p* = 0.097, Cohen’s *d* = −0.70. In all cases, physicians showed higher levels on these variables.

### Hypothesis testing

The results of the regression analyses (M1-M4) are shown in Table 2. With respect to H1, positive but non-significant trends emerged. In M1, a positive but non-significant association between problem solving and log HCC emerged at average levels of NA and controlling for sex and occupational role, *b* = 0.12, *p* = 0.09. A similar pattern was observed for overload in M3, *b* = 0.10, *p* = 0.08. Although the point estimates corresponded to a 13.1% increase in HCC for a one-unit increase in problem solving (M1) and a 10.7% increase for a one-unit increase in overload (M3), these estimates should be interpreted cautiously. Overall, H1 was not supported.

**Table 2:** Multiple regression analyses for log cortisol (N = 67).

Variable	Dependent variable: log cortisol							
	Model 1 Problem solving		Model 2 Time pressure		Model 3 Overload		Model 4 Emotional demands	
	*B*	*SE*	*B*	*SE*	*B*	*SE*	*B*	*SE*
Sex ^a^	−0.42†	0.21	−0.51*	0.22	−0.53*	0.22	−0.48*	0.23
Position held ^b^	0.72**	0.24	0.73**	0.23	0.66**	0.25	0.76**	0.24
Job demand ^c^	0.12†	0.07	0.11	0.09	0.10†	0.06	0.03	0.06
Negative affectivity	0.00	0.09	0.04	0.10	0.02	0.10	0.01	0.10
Job demands × Negative affectivity	0.21**	0.07	0.22**	0.08	0.08	0.07	0.07	0.07
*R* ^2^	0.27**		0.26**		0.21*		0.17*	
Δ*R*^2^	0.10**		0.09**		0.02		0.01	

The independent variables included in the models (excluding dichotomous variables) were mean-centered, to enable easier interpretations of the results. The values of cortisol were log-transformed prior to data analysis.

a 0 = female, 1 = male.

b 0 = nurses, 1 = physicians.

c In each model, a different job demand was analyzed. These were problem solving in Model 1, time pressure in Model 2, overload in Model 3, and emotional demands in Model 4.

^†^*p* < 0.10. *p < 0.05. **p < 0.01.

Turning to H2, the interaction between problem solving and NA was significant in M1, *b* = 0.21, *p* < 0.01, and accounted for an additional 9.8% of the variance in log HCC, *F*_change_(1, 61) = 8.25, *p* < 0.01, *f*^2^ = 0.14, a small to medium effect. Simple slope analysis showed that the association between problem solving and log HCC was positive and significant when NA was high, *b* = 0.31, *p* < 0.01, but not significant when NA was low, *b* = −0.06, *p* = 0.47. Specifically, each one-unit increase in problem solving was associated with a 36.2% increase in HCC when NA was high, whereas it was associated with a non-significant 6.1% decrease when NA was low. The interaction between problem solving and NA is shown in Figure 1.

**Figure 1:**
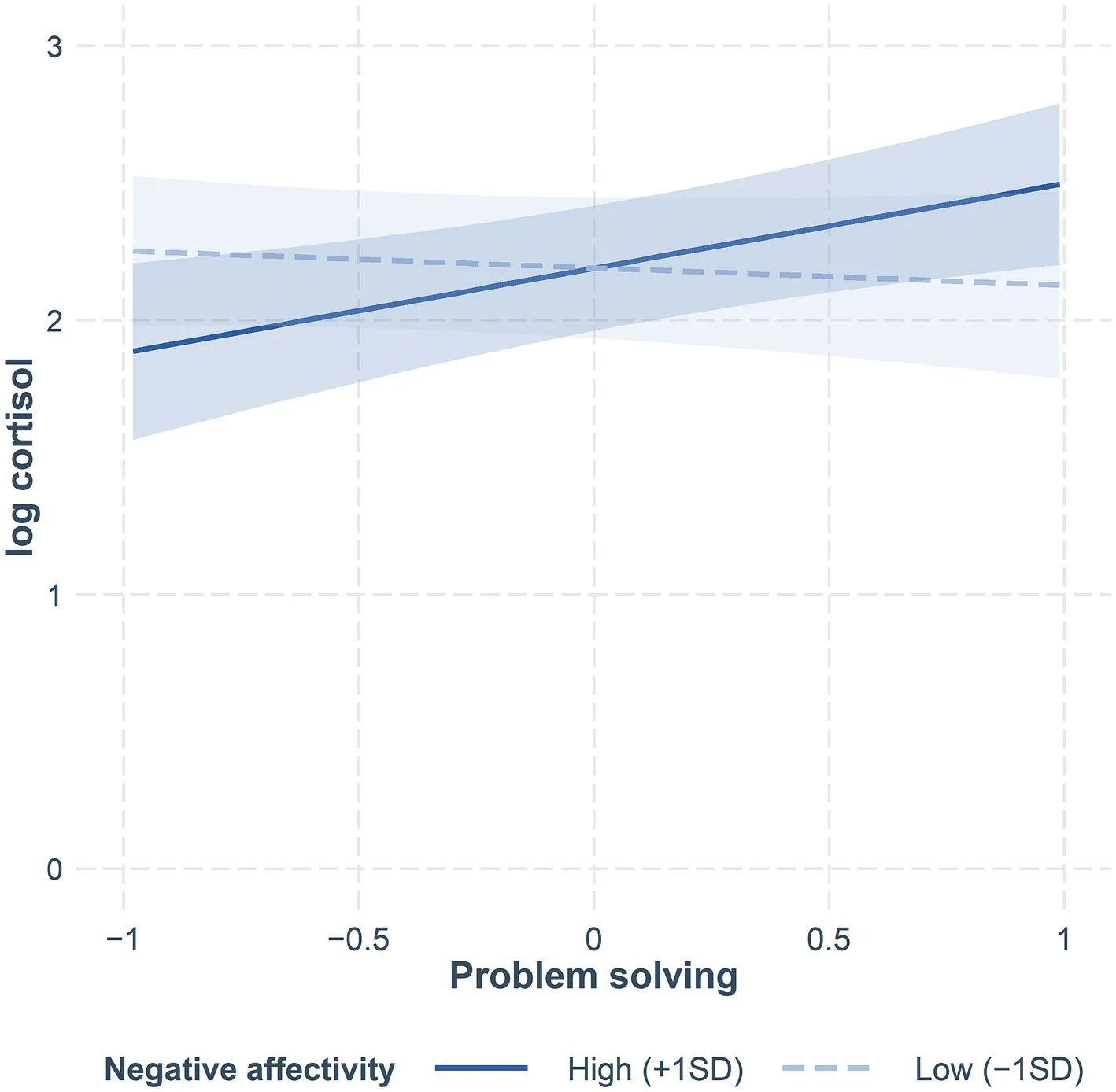
The moderating role of negative affectivity in the relationship between problem solving and log cortisol. Confidence bands represent 95% confidence intervals.

The interaction between time pressure and NA was significant in M2, *b* = 0.22, *p* < 0.01, and accounted for an additional 9.4% of the variance in log HCC, *F*_change_(1, 61) = 7.76, *p* < 0.01, *f*^2^ = 0.13, a small to medium effect. Simple slope analysis showed that the association between time pressure and log HCC was positive and significant when NA was high, *b* = 0.31, *p* = 0.02, but not significant when NA was low, *b* = −0.09, *p* = 0.36. Specifically, each one-unit increase in time pressure was associated with a 36.1% increase in HCC when NA was high, whereas it was associated with a non-significant 9% decrease when NA was low. The interaction between time pressure and NA is shown in Figure 2.

**Figure 2:**
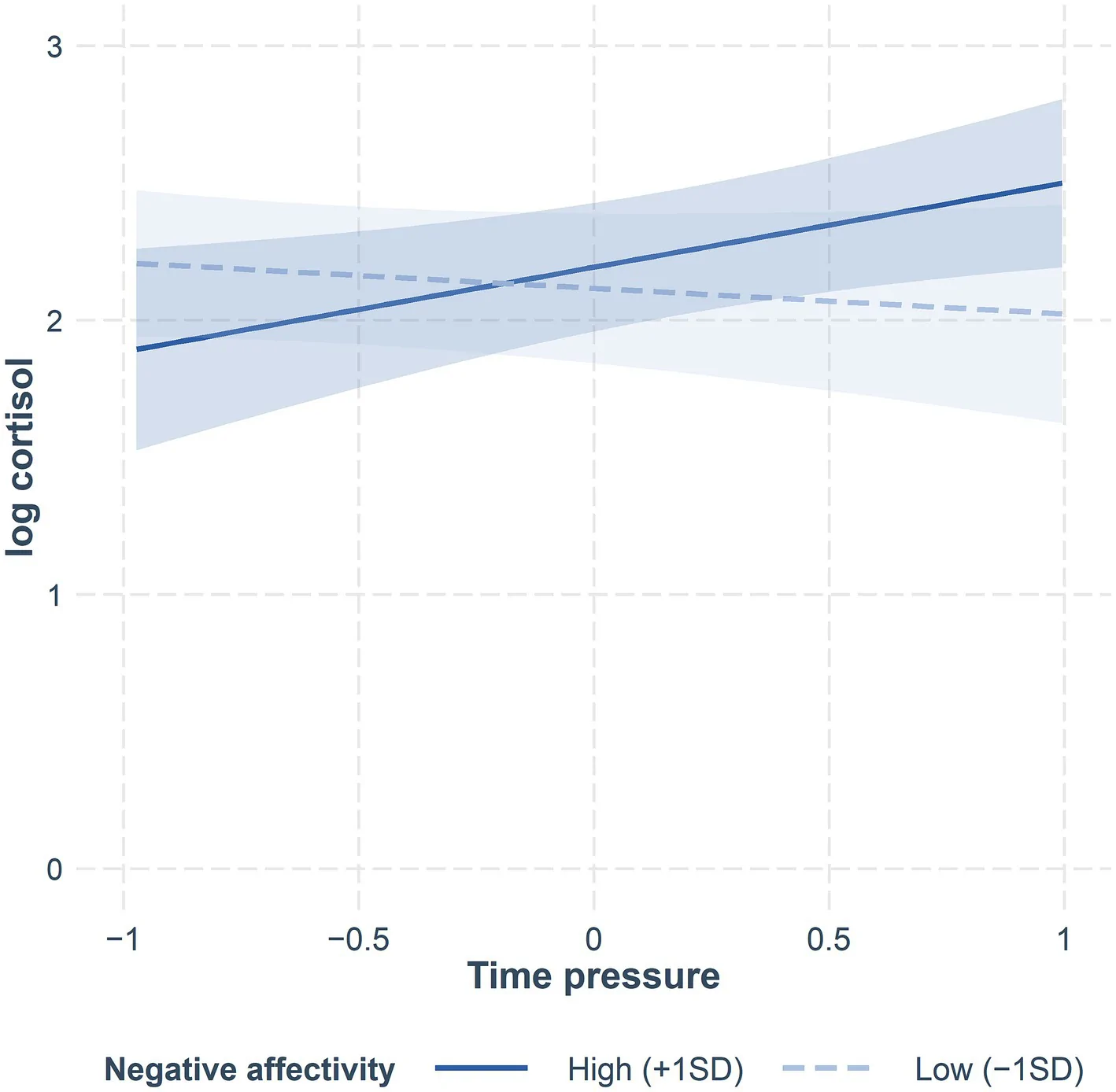
The moderating role of negative affectivity in the relationship between time pressure and log cortisol. Confidence bands represent 95% confidence intervals.

Finally, the interaction between overload and NA was not significant in Model 3, *b* = 0.08, *p* = 0.23, nor was the interaction between emotional demands and NA in Model 4, *b* = 0.066, *p* = 0.34. Hypothesis 2 was partially supported for problem solving and time pressure.

### Model diagnostics and sensitivity analyses

Additional analyses were conducted to assess the regression models and the stability of the findings (Fox and Weisberg, 2019). In all four models, residual diagnostics did not indicate departures from normality or identify outlying residuals. Visual inspection of the residual plots did not suggest departures from linearity, consistent with the nonsignificant Tukey tests. However, tests indicated non-constant residual variance in all four models. Accordingly, we computed 95% percentile confidence intervals based on 5,000 case-bootstrap resamples. To assess the influence of individual observations, we also refitted each model after excluding, one at a time, the three observations with the largest Cook’s distances.

Results of regression analyses with bootstrap confidence intervals are reported in Table 3. In M1, the interaction term was significant, *b* = 0.21, 95% CI [0.03, 0.32]. Simple slope analysis showed that the association between problem solving and log HCC was positive and significant when NA was high, *b* = 0.31, 95% CI [0.13, 0.50], but not significant when NA was low, *b* = −0.06, 95% CI [−0.21, 0.16]. In M2 the interaction term was significant, *b* = 0.22, 95% CI [0.03, 0.34]. Simple slope analysis showed that the association between time pressure and log HCC was positive and significant when NA was high, *b* = 0.31, 95% CI [0.10, 0.49], but not significant when NA was low, *b* = −0.09, 95% CI [−0.24, 0.15]. The interaction term was not significant in M3, *b* = 0.08, 95% CI [−0.05, 0.21], and M4, *b* = 0.07, 95% CI [−0.08, 0.19]. Additionally, in the case-deletion analyses, the interactions remained statistically significant in M1 and M2, whereas those in M3 and M4 remained non-significant.

**Table 3:** Multiple regression analyses with percentile bootstrap confidence intervals for log cortisol (*N* = 67).

Variable	Dependent variable: log cortisol											
	Model 1 Problem solving			Model 2 Time pressure			Model 3 Overload			Model 4 Emotional demands		
	*B*	95% CI		*B*	95% CI		*B*	95% CI		*B*	95% CI	
		*LL*	*UL*		*LL*	*UL*		*LL*	*UL*		*LL*	*UL*
Sex ^a^	**−0.42**	**−0.83**	**−0.04**	**−0.51**	**−0.98**	**−0.06**	**−0.53**	**−1.01**	**−0.07**	**−0.48**	**−0.92**	**−0.06**
Position held ^b^	**0.72**	**0.08**	**1.39**	**0.73**	**0.09**	**1.38**	**0.66**	**0.004**	**1.34**	**0.76**	**0.03**	**1.44**
Job demand ^c^	**0.12**	**0.004**	**0.26**	0.11	−0.03	0.24	0.10	−0.01	0.21	0.03	−0.11	0.15
Negative affectivity	0.00	−0.13	0.17	0.04	−0.10	0.23	0.02	−0.13	0.20	0.01	−0.15	0.19
Job demands × Negative affectivity	**0.21**	**0.03**	**0.32**	**0.22**	**0.03**	**0.34**	0.08	−0.05	0.21	0.07	−0.08	0.19

B denotes the unstandardized OLS regression coefficient. Confidence intervals are 95% percentile intervals based on 5,000 case-bootstrap resamples. Boldface indicates coefficients whose 95% percentile bootstrap confidence intervals exclude zero. The values of cortisol were log-transformed prior to data analysis. CI, confidence interval; LL, lower limit; UL, upper limit.

a 0 = female, 1 = male.

b 0 = nurses, 1 = physicians.

c In each model, a different job demand was analyzed. These were problem solving in Model 1, time pressure in Model 2, overload in Model 3, and emotional demands in Model 4.

Finally, additional analyses were conducted to assess the robustness of the results in light of the modest internal consistency of the negative affectivity measure. The first item had an adjusted item–total correlation of 0.25, below the 0.30 threshold (Boateng et al., 2018), whereas the correlations for the remaining items ranged from 0.44 to 0.55. We therefore re-estimated all four models using an NA score that excluded the first item. Internal consistency increased slightly (Cronbach’s α = 0.67 for three items). The pattern of interaction results was unchanged: the 95% percentile bootstrap confidence intervals excluded zero for the interactions involving problem solving and time pressure but included zero for those involving overload and emotional demands.

## Discussion

Based on the JD-R theory (Bakker et al., 2023; Demerouti et al., 2001) and AL model (McEwen, 1998), this exploratory cross-sectional study examined the relationship between JDs and HCC among workers in the healthcare sector, with a focus on the moderating role of NA.

With respect to the first hypothesis, problem solving and overload showed positive but non-significant associations with HCC. Therefore, although the direction of the coefficients was consistent with the expected pattern, these findings do not provide robust evidence for direct associations between these job demands and HCC. Accordingly, any interpretation of these main effects should remain cautious, and further research is needed to clarify whether prolonged or chronic exposure to specific JDs may be associated with potential dysregulation of the HPA axis reflected by increased HCC. More importantly, we observed that the association between JDs—specifically problem solving and time pressure—and HCC was stronger at higher levels of NA. This association was positive and significant when NA was high, but not significant when NA was low. Accordingly, our findings suggest that NA may function as a vulnerability factor: HCWs high in NA may be more reactive to their work environment and may exhibit a heightened strain response to certain JDs.

Some of our findings should be contextualized in light of previous literature. In particular, the lack of association between JDs and HCC—at least at average levels of NA—does not imply that these demands lack the potential to elicit psychological and/or physiological strain. Stress influences multiple interrelated biological systems that interact to facilitate adaptation to perceived threats, including the HPA axis but also the SAM axis and the immune system. Evidence from previous research suggests that exposure to stress can lead to dysregulation within any of these systems, potentially contributing to adverse health outcomes (O’Connor et al., 2021).

Second, the lack of association between emotional demands and HCC may appear unexpected, considering that interactions with patients and their families are central to the professional role of HCWs. Although emotional demands and problem solving can be conceptualized as challenge demands under certain conditions, problem solving may require sustained cognitive effort, vigilance, responsibility, and continuous decision-making, which could contribute more directly to prolonged physiological activation and HPA-axis dysregulation. By contrast, emotional demands in healthcare settings may be partly buffered by the meaningful and prosocial nature of caregiving interactions. Healthcare professionals may perceive emotionally demanding encounters not only as stressful, but also as intrinsically rewarding and aligned with professional values and identity. Consequently, emotional demands may simultaneously activate strain and motivational processes, potentially attenuating their overall association with HCC (Podsakoff et al., 2023).

We argue that this study offers a valuable contribution to the existing literature for at least two key reasons. First, by showing an interaction between specific JDs and NA, this study offers a novel perspective on the interplay between work-related and individual characteristics in influencing HCC as a physiological marker of stress. This contribution is particularly relevant, as previous research on the association between JDs—often operationalized as workload—and HCC has produced mixed findings, with evidence of positive, negative, or even null associations (for a review, see Schaafsma et al., 2021). These inconsistencies suggest that JDs may be linked to hypercortisolism, hypocortisolism, or no significant physiological effects at all (Schaafsma et al., 2021). Interestingly, a similar pattern of results has also been observed in research on job burnout (Rohleder, 2018). However, in line with transactional models of stress, this study suggests that the impact of JDs on strain responses may vary depending on individual characteristics such as NA, which influence how individuals appraise and manage stressful situations at work. It further highlights the importance of individual characteristics in shaping the stress process and influencing both psychological and physiological responses (Ganster and Rosen, 2013).

Second, although a substantial body of literature has addressed the topic, the role of NA in the stress process remains poorly understood, with limited evidence concerning its influence on physiological indicators such as cortisol (Miller et al., 2016; Quirin et al., 2009). The study contributes to the literature by showing that NA may influence physiological reactivity to (at least some) stressful situations at work, which is consistent with the “hyper-responsivity mechanism” (Spector et al., 2000) or the “differential reactivity model” (Bolger and Zuckerman, 1995). However, our findings extend these mechanisms beyond psychological outcomes (e.g., mental health, distress, job attitudes; Hochwarter et al., 2005; O’Brien et al., 2008) to include physiological outcomes—namely, elevated HCC. The stronger associations of problem-solving demands and time pressure with HCC among HCWs with high NA may reflect the use of coping strategies less suited to these demands. Previous research has associated NA with emotion-focused and avoidant coping (Eaton and Bradley, 2008), while neuroticism—a construct related to NA (Kaplan et al., 2009)—has been negatively associated with problem-solving coping (Connor-Smith and Flachsbart, 2007). Because coping effectiveness may depend on both individual characteristics and the nature of the stressor (Blaxton and Bergeman, 2017), individuals high in NA may be less inclined to use strategies such as planning, prioritization, and timely decision-making, which could be useful in managing demands like problem-solving and time pressure. Hence, problem-solving demands and time pressure could be more strongly associated with HCC among high-NA individuals. At the same time, this proposed mechanism may be less relevant to emotional demands, which may not be readily addressed through planning and prioritization, while neither avoidant coping nor emotion-focused strategies such as seeking comfort or empathy are necessarily effective strategies in reducing the costs associated with these demands. Overload, in turn, may reflect staffing constraints that individual coping cannot readily change or control. These possibilities could help explain why we did not detect interactions of NA with emotional demands or overload. However, these explanations remain speculative because coping and organizational constraints were not measured in this study, and more research is needed to test these possible explanatory mechanisms.

### Limitations and future directions

The results of this study should be interpreted in light of several limitations. First, the demographic composition of the sample—particularly the sex distribution (82.1% female)—was imbalanced. Although this distribution reflects the occupational profile of the healthcare facility (83.6% were nurses), and sex imbalance is not uncommon in studies using hair-based biomarkers (e.g., individuals who are bald or have short hair are typically excluded; Ben Assayag et al., 2017), these factors nonetheless limit the generalizability of the findings to the broader population of healthcare workers. Hence, this research should be regarded as an exploratory study aimed at generating preliminary evidence on the associations under investigation.

The small sample size may also have reduced statistical power to detect smaller effects and may limit the generalizability of the findings. To address this concern, we conducted a sensitivity power analysis (Lakens, 2022). The analysis indicated that, with *N* = 67, alpha = 0.05, and power set at 1 - β = 0.80, the study was able to detect a minimum local effect size of approximately *f*^2^ = 0.12 for the two-way interaction term. This effect size is slightly below the conventional threshold for a medium effect in multiple regression (*f*^2^ = 0.15), suggesting that the study was sufficiently powered to detect interaction effects of at least moderate magnitude. Accordingly, the non-significant interaction effects observed for overload and emotional demands should be interpreted cautiously, as effects of smaller magnitude may have gone undetected. The modest internal consistency of the negative affectivity scale may have reduced the precision of the moderation estimates, although the pattern of results remained unchanged when the item with the lowest adjusted item–total correlation was excluded. All in all, replication with larger and more diverse samples would be valuable to confirm and extend the present findings.

Given the field-based nature of the study and the privacy requirements, we were unable to collect information on participants’ age, body mass index, smoking status, and oral contraceptive use. Consequently, we were unable to account for the potential influence of these factors on HCC. Future research should seek to replicate our findings while accounting for these potential confounders (Stalder et al., 2017; van der Valk et al., 2022; Wosu et al., 2015). We also lacked information on shift work, including night shifts, which may be associated with HCC, potentially through disrupted sleep patterns (Staufenbiel et al., 2013). Cosmetic hair practices, such as hair dyeing, bleaching, and other chemical treatments, were not assessed, despite their potential influence on HCC.

Next, the cross-sectional nature of our research design limits the ability to determine the temporal sequence of the variables examined—a necessary condition for drawing causal inferences. Although HCC reflects cortisol accumulated over time (Abell et al., 2016), this research cannot establish consequences of exposure to JDs. It should also be acknowledged that job demands and negative affectivity were assessed using the same self-report questionnaire; thus, common method variance and individual differences in the reporting of demands may have influenced their observed associations. However, because HCC was assessed independently using hair samples, shared-method bias is less likely to account for its associations with the self-reported measures. Nevertheless, the present design cannot distinguish differences in working conditions from differences in how those conditions were perceived and reported. The moderation findings should therefore be interpreted as involving perceived job demands and replicated using independent assessments of job demands. Finally, our study focused exclusively on JDs, without accounting for job resources or the dynamic interplay between demands and resources, which are also known to influence strain responses according to the JD-R theory.

### Practical implications

These findings may have practical implications, although the effectiveness of any resulting interventions would need to be assessed in future research. Specifically, managing JDs with attention to individual differences in stress reactivity—particularly regarding NA—may help mitigate the physiological impact of high-pressure environments, such as those commonly found in the healthcare sector. Tailored interventions could include training to improve time management and emotional regulation skills, as well as to promote awareness of personal dispositions that influence stress perception and coping choices or effectiveness. Such programs may help HCWs with high NA to appraise work demands as less threatening and to adopt more adaptive coping strategies, thereby reducing maladaptive stress responses. Additionally, managers can play a key role in shaping how employees interpret and respond to job demands. Leadership styles that foster open communication, provide support, and offer clarity of expectations may buffer the impact of job stressors—particularly for individuals who are more emotionally reactive.

## Conclusion

In conclusion, this study provides preliminary evidence of the complex interplay between environmental and individual characteristics in shaping physiological strain responses among HCWs. Anchored in the JD-R theory and the AL model, our findings indicate that specific JDs—particularly problem solving and time pressure—are associated with elevated HCC among individuals high in NA. This underscores NA’s role as a vulnerability factor, which may play a role in modulating the physiological impact of work-related stressors. This is consistent with transactional models of stress, according to which individual characteristics may affect stressor appraisal and coping. Although these results should be interpreted with caution given the exploratory nature of the study and its limited sample size, they contribute to a growing body of research integrating psychological and biological perspectives on W-RS. From a practical standpoint, the results highlight the potential value of designing occupational health interventions that are sensitive to individual traits—such as NA—that can be associated with heightened physiological stress reactivity. In high-demand settings like healthcare, such personalized approaches may represent a useful strategy for promoting sustainable well-being and potentially mitigating long-term health risks.

## Data Availability

The raw 16S and 18S rRNA gene amplicon sequences have been deposited in the NCBI Sequence Read Archive (SRA) under BioProject accession numbers PRJNA1550242 (16S, bacterial communities) and PRJNA1550291 (18S, eukaryotic communities).
